# Low risk, high reward? Repeated competitive biddings with multiple winners in health care

**DOI:** 10.1007/s10198-019-01143-1

**Published:** 2020-01-04

**Authors:** Visa Pitkänen, Signe Jauhiainen, Ismo Linnosmaa

**Affiliations:** 1 Research Department, Social Insurance Institution of Finland, P.O. Box 450, 00056 Helsinki, Finland; 2 Department of Health and Social Management, University of Eastern Finland, P.O. Box 1627, 70211 Kuopio, Finland; 3 Centre for Health and Social Economics, National Institute for Health and Welfare, P.O. Box 30, 00271 Helsinki, Finland

**Keywords:** Health care, Competitive bidding, Competition, Choice modelling, Prices, C57, H51, I11, I18

## Abstract

We study physiotherapy providers’ prices in repeated competitive biddings where multiple providers are accepted in geographical districts. Historically, only very few districts have rejected any providers. We show that this practice increased prices and analyze the effects the risk of rejection has on prices. Our data are derived from three subsequent competitive biddings. The results show that rejecting at least one provider decreased prices by more than 5% in the next procurement round. The results also indicate that providers have learned to calculate their optimal bids, which has also increased prices. Further, we perform counterfactual policy analysis of a capacity-rule of acceptance. The analysis shows that implementing a systematic acceptance rule results in a trade-off between direct cost savings and service continuity at patients’ usual providers.

## Introduction

Public authorities in the European Union spend around 14% of the GDP on the purchase of services, works and supplies using public procurement [1]. There is an increasing trend also among health and social care organizers to use competitive elements such as competitive biddings [2]. Well-known imperfect market conditions such as uncertainty and asymmetric information characterize health care markets [3]. Therefore, several issues need to be considered when competitive biddings are used in health care. For example, it is typical to organize the competitive biddings in a repeated manner and select multiple service providers for each contract period. Meanwhile, many patients may receive the service continuously and prefer to visit their usual provider. Competitive biddings give information about the right price-level, but the procurement may also change service providers and end long-lasting relationships between patients and providers. Even though these properties are well-known, only very few studies have analyzed the properties of competitive biddings in health care.

In this study, we analyze competitive biddings that were organized by the insurance districts of the Social Insurance Institution of Finland (Kela) in 2003, 2006, 2010, and 2014 to acquire providers for physiotherapy service for disabled individuals. The districts were responsible for acquiring multiple providers based on their quality–price ratio and the local demand for the service. However, the districts did not have strict budget constraints and historically only very few districts rejected any providers. This setting provides an interesting opportunity to examine the properties of repeated competitive biddings with multiple winners in health services. In the main analysis, we study the effects of the risk of rejection on prices. We also perform counterfactual policy analysis, to examine the direct fiscal effects of implementing a systematic capacity-based rule of provider acceptance.

First, we present descriptive evidence and show that the overall price-level of the bids for a 45-min physiotherapy service increased from an average of 33 euros in 2003 to 58 euros in 2014. Meanwhile, the highest bid increased from 55 to 116 euros. Then, we examine more closely the effects that the risk of rejection had on prices in the 2010 and 2014 competitive biddings. Our main data sources are quality–price scoring tables collected from the insurance districts, merged with patient-level register data on patients’ choice of provider in 2006–2015. We measure the risk of rejection using geographical variation in the insurance districts’ rejection rates in previous competitive biddings. We also analyze whether providers’ previous distance to the rejection threshold had an effect on their prices. In our analysis, we control for market competition, using measures that are based on the value that each provider brings into the insurance district’s network, as well as several other provider and area-level factors.

The results show that rejecting at least one provider decreased prices by more than 5% in the next competitive bidding. The effect is stronger in 2014 than 2010, which suggests that the providers learned the institutional practices of the procurement procedure. We also find that providers which were located further from the rejection threshold increased their prices more heavily in the next competitive bidding. This indicates that providers learned to calculate their optimal bids, which further increased the overall price-level. These results suggest that implementing a systematic capacity-rule of acceptance in each district would have lowered the overall price-level of the service. We simulate the effects of counterfactual scenarios where providers are accepted based on their annual capacity and the local demand for the service. The analysis shows that the regulation would have decreased the costs especially in the 2014 procurement. However, many patients would have been forced to switch their usual provider, which is problematic in a service that is based on continuous relationships between patients and providers.

The study is related to several distinct strands of literature. First and foremost, the study is related to the use of competitive biddings in health care. We are the first to provide empirical analysis regarding competitive biddings in a health service, which patients receive continuously and for which multiple service providers are acquired in repeatedly organized procurements. Theoretical literature suggests that competitive bidding can be a powerful mechanism to decrease the health care expenditure, but their design and implementation must be done carefully [4, 5]. Empirical literature on competitive biddings in health services is very scarce and comes mainly from the analysis of competitive biddings in the US Studies have shown that insurers can use their market power for higher bids in the competitive biddings of Medicare [6, 7]. Similarly, the results of this study show that price bids have been higher in less competitive areas.

The study also contributes to the literature on the effects of competition on prices in health care. For this purpose, the Finnish physiotherapy markets offer an excellent setting because the market consists of a large number of small private providers. Most of the previous literature has documented that competition decreases prices in hospital services [8]. Pekola et al. [9] analyzed the effects of competition on quality and prices using the same setting with a sample of providers in the 2010 competitive bidding. They found that competition had a weak negative effect on quality but no effect on prices. This study extends their work by taking more precisely into account the historical and institutional setting as well as using a much richer provider and patient-level data.

The study also relates to provider contracting in health care and the side-effects of narrowing the network from which patients can choose their provider. Higuera et al. [10] show that narrowing the network can reduce costs, but patients are willing to pay for a wider network that includes their usual provider. Similarly, our study illustrates the simple trade-off between economically efficient procurement and continuity of care at the patients’ usual provider. Finally, the study provides evidence regarding patient choices and provider quality. Our results are similar to previous literature [11–12], showing that physiotherapy patients choose large, high-quality providers within short distances.

## Competitive biddings in health care

Many countries have implemented policies that increase competition among health care providers [13]. The main purpose of the reforms has been to improve the efficiency and quality of the services. Three main types of provider competition have been presented in the health economics literature: competition in a market, competition for a market and yardstick competition [2]. This paper considers a case where competition both in and for a market is present. Competition in a market usually means that providers compete with quality attributes to attract patients, and the money follows the patients to their selected provider. In this case, prices are often fixed, and the organizer needs to determine the appropriate price-level. To choose, patients should have several alternative providers and access to quality information. Nevertheless, patient choice has become very common in primary health services in European countries [14], and there is increasing empirical evidence showing that quality influences patient choices [11, 15].

Competition for a market means that several potential providers compete for the right to provide services or goods. The purchaser selects one or more providers in, for example, a geographical area. Common examples include the purchasing of pharmaceutical products or hospitals competing to be included in an insurer’s network of providers. Competition for a market requires that the service organizer can describe the services or goods in an accurate and verifiable way [2]. Providers and their prices can be determined, for example, using competitive bidding or bargaining between the purchaser and provider. Compared to fixed prices, competitive bidding provides information about both the prices and the providers that deliver the services at the lowest prices [16]. Therefore, competitive biddings have gained an increasingly important role in discussions about the future financing of health care services [7, 17].

Public procurements and auctions often work very well, but their design must be sensitive to the details of the institutional setting [18]. According to McCombs and Christianson [19], competitive bidding where multiple providers are selected has following four main advantages: first, selecting more than one provider gives flexibility and ensures service availability in cases where a provider exits the market or the demand suddenly increases during the contract period. Second, it might encourage more providers to participate in the procurement, because it increases the probability of being included in the pool of providers. Third, it provides patients a larger degree of choice from the pool of providers, which in turn might increase quality competition in the market among providers. Fourth, accepting multiple providers might ensure market competitiveness in subsequent competitive biddings by preserving viable competitors.

McCombs and Christianson [19] also point out three potential disadvantages: first, in areas where the number of potential providers is very small, there are only weak incentives to submit low bid prices, because the probability of losing is very low. Second, adverse selection problems under a per-episode reimbursement scheme may occur, because the price does not vary but some patients may require more intensive care. Therefore, some providers might try to avoid taking the most demanding patients. Third, accepting a large number of providers might decrease providers’ expectations of number of potential patients and gains from economies of scale, reducing the number of low price bids.

McCombs and Christianson [19] also discuss how the line between accepted and rejected providers should be drawn. A potential procedure is to use a capacity-based rule of acceptance, which requires that all providers submit a maximum capacity of service they can provide during the course of their contract. Accepted providers are then selected in an ascending order of bids, based on the possible quality–price rule, until the target capacity is reached. Under these conditions, the procurement results in a number of providers that ensures access to the services and the long-run market competitiveness. However, if a strict and clear rule of acceptance is not used, providers have strong incentives to “game” the system by submitting bids that are higher than their costs but lower than their estimate of the lowest possible rejected bid.^1^ McCombs and Christianson [19] hypothesize that the “gaming” will eventually increase the overall price-level of the bids. Our study empirically tests this hypothesis.

## Institutional setting

The institutional setting of the study is an individual outpatient physiotherapy service, which is part of the intensive medical rehabilitation services financed and organized by Kela. The physiotherapy is intended for disabled persons under 65 years of age, who face problems managing daily activities and fulfill the criteria defined by law. The basis of the service is a written rehabilitation plan that is drawn up with a physician for 1–3 years at a time. Patients do not pay any out-of-pocket payments and have a free choice of provider from the pool of accepted providers. Typically, patients receive sessions that last for 45 or 60 min, a couple times a week for several years. The physiotherapy is received either at the provider’s facilities or at patient’s home. 14,756 patients received the service in 2015, and the total costs were 73.5 million euros [21].

Kela acquires the service from private physiotherapy providers. Physiotherapy markets are among the most competed health services in Finland. Based on Statistics Finland’s registers there were 2632 independent physiotherapy providers that had 3655 employees and annual turnover of 302 million euros in 2015. Meanwhile, Kela purchased the service from 1253 different providers in 2015. Thus, around 48% of all physiotherapy providers in Finland are Kela’s service providers and the service covers around 24% of the sector’s annual turnover. Overall, Kela is the largest single financer of different rehabilitation services in Finland. Other large purchasers of physiotherapy services for different patient groups are municipalities, the occupational health care sector and insurance companies. Compared to the physiotherapy services organized by municipalities and other financers, patients who receive the service organized by Kela have more severe disabilities and require more intensive therapy.^2^ Individuals can also visit private physiotherapists by paying out-of-pocket payments.^3^ Usually patients do not simultaneously receive the rehabilitation services that are financed by different organizations.

**Fig. 1:**
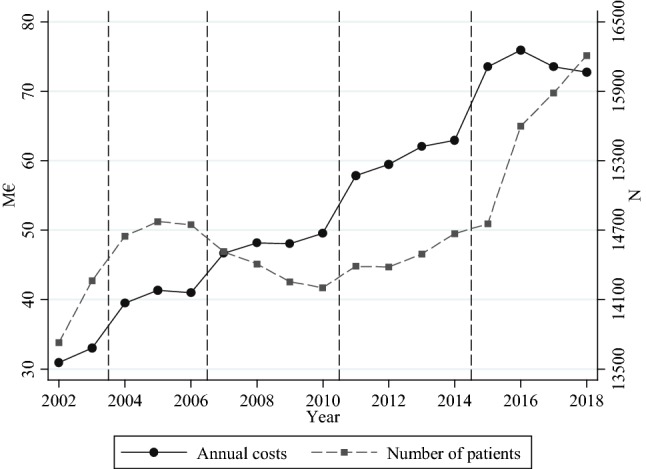
Number of patients and annual costs of the service in 2002–2018. Vertical dashed lines present a change in the contract periods. Presented costs are nominal

Kela’s insurance districts are responsible for organizing the service for the local population. The districts acquire the service from private physiotherapy providers using a competitive bidding,^4^ which they have organized in a similar and predefined manner since 2003.^5^ Most of the districts negotiated prices directly with the providers prior to 2003.^6^ The three latest competitive biddings were organized in 2006, 2010 and 2014 for the contract periods 2007–2010, 2011–2014, and 2015–2018. Figure 1 presents the annual nominal costs of the service and the number of patients in 2002–2018. The figure shows that while the number of patients remained relatively stable before the latest contract period, the annual nominal costs more than doubled. The largest cost increase took place in the first year of each contract period, when new prices were set. The number of patients has increased by around 10% during the latest contract period. However, there has been no increase in total costs. This indicates that the annual number of physiotherapy sessions per patient has decreased, most likely as a result of financial pressures caused by the rising costs.

The procurement process begins with a request for tenders. Providers provide information on their quality, annual capacity and set a price for a 45-min physiotherapy session in their tenders. The tenders are submitted to the insurance district where the providers are based. The districts evaluate the tenders and rank the providers that meet the minimum criteria based on their quality–price scores.^7^ Each district decides a rejection threshold based on the capacity of the providers and estimated local demand for the service. The district manager approves this threshold. Providers above the threshold are offered a 4-year contract, and providers that sign the contract form a pool of providers from which patients can freely choose their provider. However, quality information on the providers is not publicly available. Providers are paid for patients’ visits based on their accepted prices. Since the 2006 competitive bidding, the districts have sent the quality–price ranking lists to each provider that submitted a tender in that district.^8^ Providers were also always given information about the quality score rules, and they were able to calculate their points from the information they provided to the district. Figure 3 (in the Appendix) presents an example of the quality–price score table that was used in the Espoo district in 2014.

**Fig. 2:**
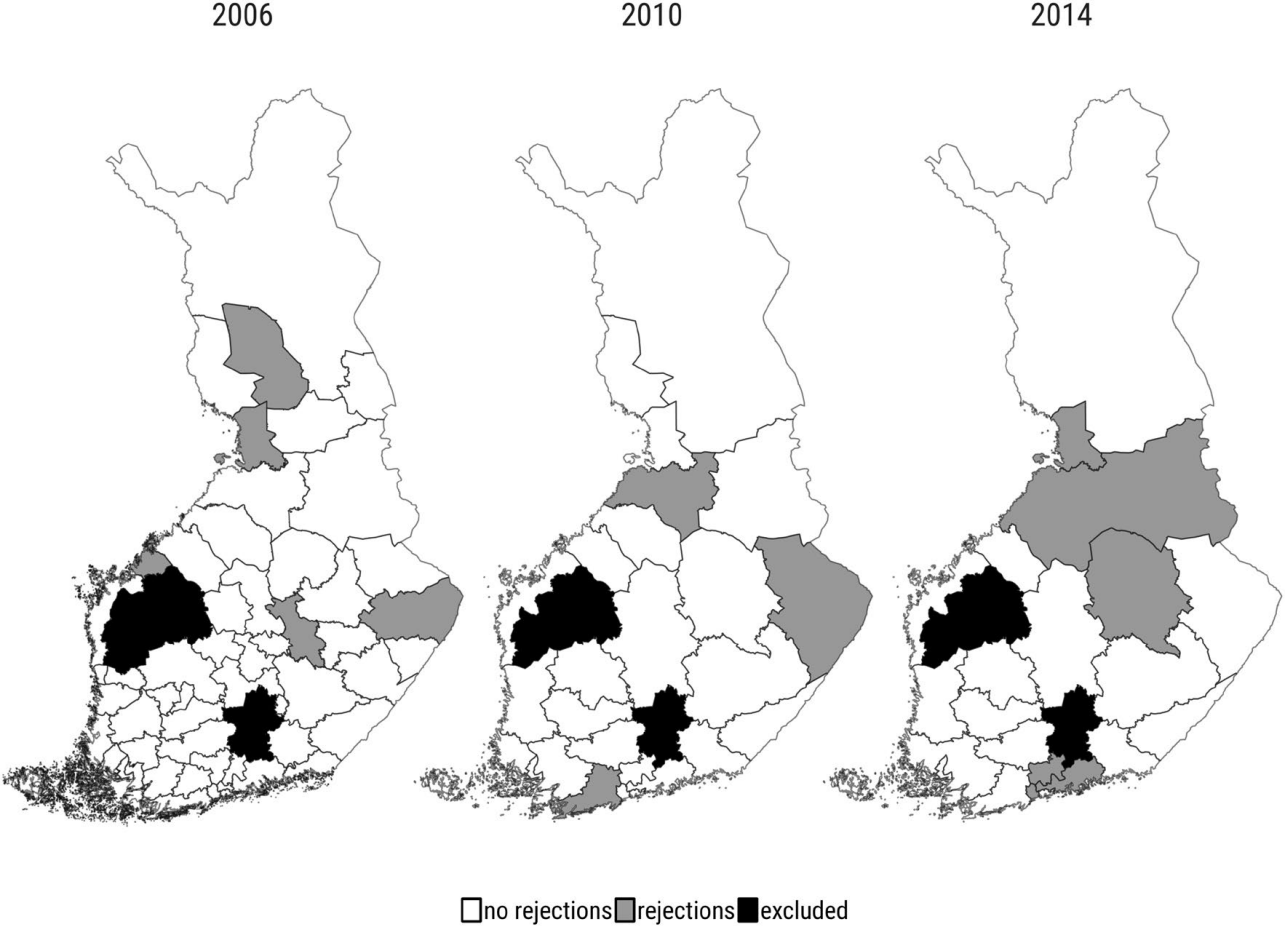
Rejections across the districts in the 2006, 2010 and 2014 competitive biddings. A price regulation pilot took place in South Ostrobothnia and Päijät-Häme districts in the 2011–2014 contract period. Therefore, providers in these districts did not participate in the 2010 competitive bidding and have been excluded from our analysis

Even though the districts were expected to accept providers based on local demand and providers’ capacity, the most common rejection threshold has been below the provider ranked last. Figure 2 shows the districts that rejected at least one provider in 2006, 2010 and 2014. In 2006 there were five districts that rejected 11 providers, in 2010 only three districts that rejected seven providers and in 2014 six districts that rejected 28 providers. Altogether only 1.2% of the providers got rejected in the three studied competitive biddings (46 out of 3769). Most of the rejected providers were single outliers, which means that they either offered a significantly high price or had a very low quality. For most of the providers the risk of being rejected was very low, which enabled them to raise prices after realizing this institutional practice. All districts acquired multiple times their required capacity in every competitive bidding. For example, in 2014 the total capacity of the accepted providers was 50,917 whereas only 14,671 patients received the service.

**Fig. 3:**
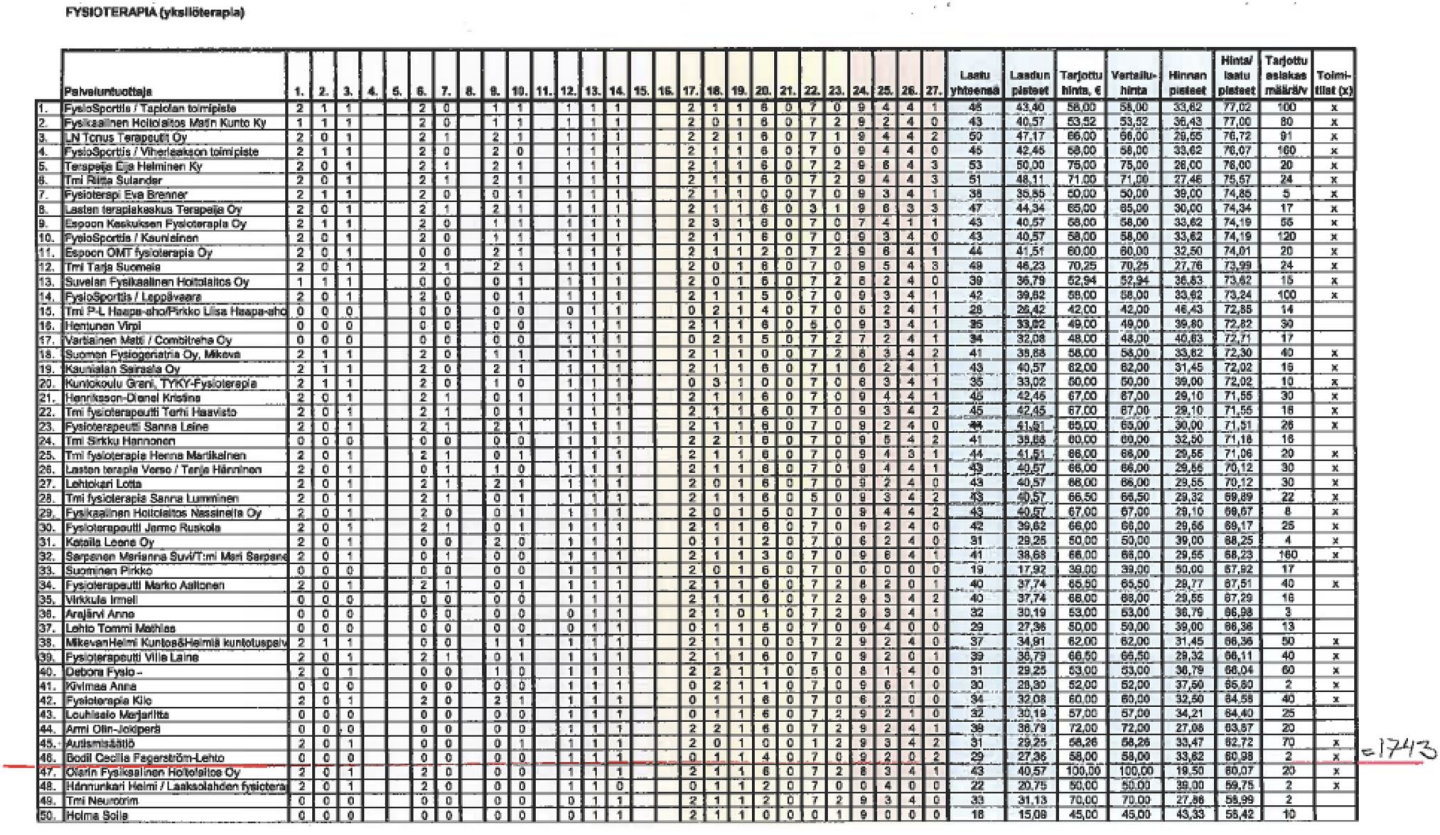
An example of the quality–price score table in the Espoo district in the 2014 competitive bidding. The red line presents the rejection threshold that the insurance district implemented based on the quality–price scores and the local demand for the service. The district accepted 46 providers as service providers with a total capacity for 1743 patients. Providers below the threshold were not offered a contract

Based on interviews with Kela procurement personnel, there were four main reasons why the districts did not reject more providers: first and foremost, Kela is obliged by law to organize the service nationwide for all eligible patients, and the districts have needed to ensure the availability of the service also in rural parts of the country. Second, the district managers did not have a budget constraint or price limit when they decided on the threshold. Third, Kela has emphasized patients’ freedom to choose from a large pool of providers. Finally, the districts have wanted to avoid a situation where a large number of patients are forced to switch providers because their previous provider was not accepted in the new pool of providers. In conclusion, the district managers did not have proper incentives to reject providers because no financial pressure existed, and rejections would have likely resulted in an increase in administrative work and negative feedback.

## Data

### Provider-level data

Our main provider-level data sources are quality–price score lists that were collected from insurance districts that organized the competitive biddings in 2006, 2010 and 2014. The lists include quality scores, price bids for 45-min service and annual capacities of all providers that fulfilled the minimum requirements and were ranked based on their quality–price scores.^9^ The data include information on the rejection threshold that each insurance district decided upon. We also have data on providers’ accepted prices in the 2003 competitive bidding, which was the first procurement organized similarly in all districts.^10^ Our data also include the providers’ address, postcode and business type information.

Measuring provider quality is challenging because quality is very multidimensional in health care [22]. Quality measures are often based on inputs such as number of staff per beds, or outcomes such as 30-day mortality. The districts measured physiotherapy providers’ quality through their investments in three main categories: education, experience and facilities. Certain other minor issues, such as language skills, also factored in. As in Forder and Allan [23], our measure of quality can be seen as a proxy for providers’ underlying performance quality or utility gain construct.

Kela implemented a fixed-price pilot in two insurance districts (South Ostrobothnia and Päijät-Häme) in the 2011–2014 contract period. We have excluded these two districts from our data and analysis. This should not result in any bias because the districts are responsible for organizing the service only for the local population and they can be considered as independent geographical entities. In total, our final data include information about 5133 bids from four consecutive competitive biddings. The descriptive statistics of the accepted and rejected providers in the studied competitive biddings are presented in Table 1.

**Table 1:** Descriptive statistics of the providers in 2003, 2006, 2010 and 2014

Variable	Accepted providers					Rejected providers				
	*N*	Mean	SD	Min	Max	*N*	Mean	SD	Min	Max
2003										
Price (€)	1364	33.41	4.52	20	55	–	–	–	–	–
2006										
Price (€)	1369	39.13	5.82	21	71	11	41.95	8.00	30	55
Quality	1283	67.13	12.06	28	103	11	58.18	16.58	24	78
Quality–price score	1283	77.07	8.46	44.38	100	11	71.83	12.58	54.71	100
Capacity	530	17.50	20.19	1	200	–	–	–	–	–
Premises	1369	0.91	0.29	0	1	11	0.73	0.47	0	1
New provider	1369	0.18	0.39	0	1	11	0.55	0.52	0	1
Patients	1369	8.81	12.86	0	152	11	2.45	4.16	0	13
ΔPrice (€)	1119	5.50	3.49	– 21	32.9	5	6.1	6.19	2	17
2010										
Price (€)	1195	47.54	7.62	28	99	7	54.07	10.22	35	68.5
Quality	1195	80.46	13.63	31	104	7	68.29	16.01	44	84
Quality–price score	1195	79.15	8.14	40	100	7	70.37	5.86	59.7	76.4
Capacity	1195	33.77	43.40	1	420	7	40.14	67.95	5	192
Premises	1195	0.92	0.27	0	1	7	0.86	0.38	0	1
New provider	1195	0.14	0.35	0	1	7	0.57	0.53	0	1
Patients	1195	10.19	14.10	0	161	7	1.14	2.61	0	7
ΔPrice (€)	1013	8.12	5.05	– 27.5	32	2	10	4.24	7	13
2014										
Price (€)	1159	57.83	9.43	34	102.5	28	70.38	16.80	45	116
Quality	1159	37.41	7.45	9	55	28	26.57	7.39	14	43
Quality–price score	1159	73.45	7.69	41.88	100	28	57.15	5.45	39.68	67.17
Capacity	1159	40.99	53.73	1	450	28	13.64	13.83	2	70
Premises	1159	0.92	0.27	0	1	28	0.71	0.46	0	1
New provider	1159	0.18	0.38	0	1	28	0.61	0.50	0	1
Patients	1159	10.85	16.08	0	179	28	1.29	2.73	0	12
ΔPrice (€)	923	10.07	5.80	– 16	54.5	11	15.62	12.80	1.58	40

ΔPrice includes providers that submitted a bid also in the previous round. Providers in the districts where a price regulation pilot took place in 2011–2014 are excluded from the data

### Patient-level data

The patient-level data are based on Kela’s registers on rehabilitation applications and invoices regarding patients who received the service in 2006–2015. The applications data contain patients’ age, sex, municipality, postcode and ICD–10 codes of primary, secondary and tertiary illnesses. The data also include information on whether the patient had the right to receive the service at home, the number of annual physiotherapy sessions and the length of the sessions in minutes. We have merged the applications data with invoices. Providers were instructed to invoice Kela once a month, and therefore the data include on average 10 invoices for each patient. The invoice data also include information on whether the patient received the service at home, based on whether a provider was paid extra for travelling. We have calculated an annual cost per patient based on the selected provider’s price, the number of annual sessions and whether the service was received at home or at the provider’s facilities. Similar to our provider-level data, we have excluded all patients who resided in the two districts where the fixed-price pilot was implemented in the 2011–2014 contract period. Table 2 presents the descriptive statistics of the patients and their choice sets in 2011 and 2015.

**Table 2:** Descriptive statistics of patients in 2011 and 2015

Variable	2011					2015				
	*N*	Mean	SD	Min	Max	*N*	Mean	SD	Min	Max
Age	12,728	38.61	20.35	0	66	13,045	37.42	20.42	0	66
Male	12,728	0.51	0.50	0	1	13,045	0.51	0.50	0	1
Sessions at home	12,728	0.40	0.49	0	1	13,045	0.54	0.50	0	1
Number of sessions	12,728	56.80	25.83	5	150	13,045	54.19	25.55	5	150
Length of a session (min)	12,728	57.47	7.22	30	120	13,045	57.61	7.10	30	90
Number of illnesses	12,728	1.69	0.81	1	3	13,045	1.89	0.85	1	3
Provider's quality	12,728	87.69	10.69	31	104	13,045	41.50	5.98	9	55
Distance to provider (km)	12,728	11.18	13.65	0.5	177.29	13,045	12.21	15.69	0.5	190.18
Provider in same district	12,728	0.91	0.28	0	1	13,045	0.90	0.29	0	1
Providers in same district	12,728	50.23	16.96	4	84	13,045	56.46	17.66	4	96
Providers in choice set	12,728	125.88	86.87	4	335	13,045	128.08	78.36	4	359
Annual costs (€)	12,728	4139	2358	275	17,280	13,045	5124	3034	287.5	25,888
Total costs (€)	52,676,311					66,847,118				

Patients in the districts where a price regulation pilot took place in 2011–2014 are excluded from the data

### Other data sources

We use open postcode data from Statistics Finland and have calculated straight-line distances between patients’ and providers’ postcodes. The calculations are based on the distances between the centre points of the postcodes.^11^ We also use Statistics Finland’s postcode-level register data that include the number of private physiotherapy providers in each postcode in 2006–2015, as well as data on average rents in each postcode in 2010–2015 that is based on Kela’s housing benefit registers for free market rents. Finally, we also have municipal-level register data on the number of individuals who were eligible for Kela’s rehabilitation services for disabled persons in 2006–2015.

## Empirical approaches

### Measures of risk

Each provider faces a risk of rejection in a competitive bidding when the procurer uses systematic acceptance criteria, for example based on local demand and providers’ capacity. However, as explained in Chapter 3 and illustrated in Fig. 2, Kela insurance districts did not apply a systematic acceptance rule. This institutional feature gives us a possibility to use the variation across the insurance districts’ rejections in the previous competitive biddings, to measure the risk of rejection. We introduce two different measures of risk. First, we create a binary variable “rejections”, which receives a value 1 if the provider is located in a district that rejected at least one bid in the previous competitive bidding, and 0 otherwise. The measure indicates whether the providers have learned to anticipate the possibility for rejections in their district, as previous quality–price scoring tables were publicly available. At least one provider was rejected only in five districts out of the 53 districts in 2006, whereas in 2010 rejections were made only in three districts out of the 27 districts.

When a profit-maximizing provider decides on the price it bids, the provider will most likely make some assumptions about the highest price it could bid, considering its quality score, and still be accepted.^12^ The provider can learn its optimal price afterwards, when it receives the quality–price score table from the district. As our second measure of risk, we exploit this feature and calculate providers’ distance to the rejection threshold in the previous competitive bidding, measured in quality–price scores.^13^ The distance to the threshold is measured to the lastly accepted provider, also in the districts where all providers were accepted. The intuition behind the measure is following: providers that were highly ranked (far above the threshold) could have bidded a higher price, and therefore might be able to take more risk in the next competitive bidding. In turn, providers that had a relatively low rank (near the threshold) or were rejected might take less risk in their next price bid.^14^ This measure only applies to providers who also participated in the previous competitive bidding, as they were able evaluate their optimal bids compared to other providers.

The decisions about the acceptance thresholds in each district are made by local Kela officials. Also, a provider’s rank in the quality–price table is influenced by all other bids in the district. Therefore, we consider both the “rejections” as well as the “distance to the threshold” variables to be exogenously determined for a single provider. One potential source of endogeneity could be that Kela officials in geographically small districts with a large number of potential service providers could have rejected providers more easily. Table 8 (in the Appendix) shows descriptive statistics regarding the districts with and without rejections in the 2006 and 2010 competitive biddings. Based on the data, there seems to be no systematic difference between the districts that rejected bids and those that did not. In particular, the statistics prove that there was no systematic quality–price threshold used in the districts. In addition, correlations between rejections and geographical size (*r* = 0.02) or the number of bidders (*r* = 0.13) are very small at the district-level.

Descriptive statistics regarding the two measures of risk are shown in Table 3. In the 2010 competitive bidding 12% of the providers were located in an area where at least one rejection was made previously, and in 2014 the proportion was 9%. The average distance to the rejection threshold was 12.7 quality–price points in 2010 and 18.3 points in 2014.

**Table 3:** Measures of risk and competition in the 2010 and 2014 competitive biddings

Variable	2010					2014				
	*N*	Mean	SD	Min	Max	*N*	Mean	SD	Min	Max
Measures of risk										
Rejections	1202	0.12	0.32	0	1	1187	0.09	0.29	0	1
Distance to threshold	964	12.76	7.40	0	42.64	934	18.31	9.51	– 9.10	49
Measures of competition										
Predicted HHI	1202	455.38	274.93	233.72	2608.42	1187	413.51	308.74	165.79	3208.39
Actual HHI	1202	537.62	248.43	239.78	1840.90	1187	552.07	348.66	203.58	3310.22

Measures of risk are based on lagged values from the previous competitive bidding

### Measures of competition

Greater competition might decrease prices also in competitive biddings. Because there is no generally agreed measure of market structure, we use two measures to show that our results are robust across the main approaches used in the previous empirical literature. We calculate Herfindahl–Hirschman Indexes (HHI) that are based on providers’ actual and predicted market shares within their insurance district. We transform both indexes into a negative natural logarithm, which eases the interpretation of the results, as –ln(HHI) increases with more competition. The index is calculated in a district *d* at year *t* in the following way:

1 $$- {\ln}({\text{HHI}}_{dt} ) = - {\ln}\mathop \sum \limits_{j = 1}^{j} \left( {\frac{{n_{j} }}{{N_{d} }}} \right)^{2} ,$$

where *n*_*j*_ is the number of actual or predicted patients at provider *j* and *N*_*d*_ is the number of patients in a district *d*.

Defining markets with geographic boundaries often has its problems as postcodes are likely to be too small and some other boundaries too large [24]. Our preferred definition of the relevant market is the insurance district, because they were responsible for organizing the service for the local population and accepting the providers in the competitive biddings. However, measures of market structure based on concentration and geographical boundaries suffer from well-known endogeneity issues in price regressions. For example, the location of the providers and of new market entrants might be associated with prices. Also, higher quality providers may attract more patients and have higher market shares, resulting in a higher HHI for their market. Because these providers usually also have higher prices, this can lead to an estimated positive relationship between price and concentration measure driven by omitted quality scores rather than by market power [25]. This should not be a problem in our setting, as we are able to control for providers’ quality in our price regressions.

There might be also other issues that are related to both competition and prices that we cannot fully control. Kessler and McClellan [26] have provided the most common strategy to mitigate the endogeneity bias. They estimate a choice model to predict patient flows among providers, and calculate market concentrations using these predicted rather than actual patient flows. We follow their strategy and calculate –ln(HHI) that is based on providers’ predicted market shares in their insurance districts. This approach to measuring competition has also been used previously [27, 28]. Descriptive statistics regarding the competition measures are also presented in Table 3. In the price regressions we prefer to use the measure of competition based on predicted flows, and the results based on actual flows are shown in the Appendix.

### Patient choice modelling

We begin the calculations of predicted patient flows by estimating choice models on patient choices in 2011 and 2015. We use a standard random utility choice model by McFadden [29] and assume that patients are rational and maximize their utility when choosing a provider. The relative utility for a patient *i* at provider *j* at time *t* is described as:

2 $$U_{ijt} = V_{ijt} + e_{ijt} = \beta_{q} Q_{jt} + \beta_{d} D_{ij} + \beta_{{(d^{2} )}} D_{ij}^{2} + \beta_{c} C_{jt} + e_{ijt} ,$$

where ${V}_{ijt}$ represents the observable utility, which depends on the provider’s quality ${Q}_{jt}$, distance ${D}_{ij}$, squared term of distance ${D}_{ij}^{2}$, and capacity ${C}_{jt}$. We allow patients’ preferences to vary according to observed characteristics such as their age, gender and rehabilitation background. The marginal utility of quality for patient *i* is:

3 $$\beta_{qi} = \beta_{q} + \beta_{q} X_{i} {^{\prime}},$$

and similar for distance and capacity.

Patients choose from a set of alternative providers ${N}_{jt}$. We have created choice sets that include all accepted providers in the patients’ insurance districts and all other providers within 80 kilometres. Provider *j* is chosen if it results in the highest utility in the choice set. We assume that the error term ${e}_{ijt}$ is independently and identically distributed (IID) with a type-1 extreme value distribution, which leads to a conditional logit model where the probability that a patient *i* selects provider *j* is:

4 $${\text{Pr}}_{{ijt}} = ~\frac{{{\text{exp}}\left( {V_{{ijt}} } \right)}}{{\mathop \sum \nolimits_{{j' \in M_{{it}} }} {\text{exp}}\left( {V_{{ij^{\prime}t}} } \right)}}$$

The results of the patient choice models for years 2011 and 2015 are found in Table 4. In general, our main results are very similar to previous empirical literature on patient choice [11–12], showing that patients prefer large, high-quality providers within close distance. The patients’ heterogeneity is captured through the interaction terms in the model, indicating that older patients are not as sensitive to quality differences between providers and prefer shorter distances. We also find that patients who receive the service at their home choose providers from longer distance, which is intuitive as they do not bear the cost of extra travel-time. The results also provide an important policy-relevant point regarding the studied market: Even though the districts did not stimulate competition for the markets by rejecting a sufficient number of providers, patient choice has encouraged quality-competition among the selected providers in the market.

**Table 4:** Conditional logit models of patient choices in 2011 and 2015

Variable	2011		2015	
	Est	SE	Est	SE
Main effects				
Quality	0.085	0.004***	0.130	0.007***
Distance	– 0.144	0.005***	– 0.120	0.004***
Distance^2^	0.0004	0.000***	0.0001	0.000***
Capacity	0.005	0.0005***	0.004	0.000***
Interaction with quality				
× Age	– 0.001	0.000***	– 0.001	0.000***
× Male	– 0.003	0.002	– 0.007	0.003*
× Number of annual sessions	– 0.000	0.000	0.0001	0.0001
× Number of illnesses	– 0.0001	0.001	– 0.003	0.002
× Sessions at home	0.005	0.002*	– 0.006	0.004
Interaction with distance				
× Age	– 0.001	0.000***	– 0.0005	0.0001***
× Male	0.002	0.002	0.002	0.002
× Number of annual sessions	0.0002	0.0001**	– 0.0001	0.0001
× Number of illnesses	0.008	0.001***	0.005	0.001***
× Sessions at home	0.018	0.003***	0.019	0.002***
Interaction with capacity				
× Age	0.0001	0.000***	0.0001	0.000***
× Male	0.0001	0.0002	0.0001	0.0002
× Number of annual sessions	0.000	0.000	0.000	0.000
× Number of illnesses	– 0.0002	0.0002	– 0.0002	0.0001
× Sessions at home	– 0.003	0.000***	– 0.002	0.000***
Number of patients	12,728		13,045	
Number of observations	1,602,202		1,671,001	
BIC	70,544.97		76,009.07	
Pseudo *R*^2^	0.400		0.378	

Estimated coefficients are marginal utilities. Interactions on patient characteristics with distance^2^ are not reported (available from the authors)

*p < 0.05; **p < 0.01; ***p < 0.001

We use the estimates of the choice models and predict provider *j*’s market share in its district in the year of the competitive bidding, by summing up patients’ estimated choice probabilities for choosing provider *j* in 2010 and 2014. We use the estimates from the 2011 choice model for predicted choices in 2010 and similarly estimates from the 2015 model for predicted choices in 2014. For the predictions we use choice sets that cover all providers in patient *i*’s insurance district. We measure the goodness-of-fit of our model by comparing the predicted results against patients’ actual choices. We follow previous literature [30–32] and calculate a “hit-or-miss” variable where predicted choice for a patient is the provider that has the greatest predicted probability. This analysis shows that our model correctly predicts 28.0% of the choices in 2011 and 26.6% in 2015. These prediction rates are comparable to the previous studies. As the demand model predicts choices well, the correlation between the HHI based on actual and predicted patient flows (*r* = 0.670) is also strong.

### Price equations

We estimate two different linear regression models to analyze the effects of risk on prices. The first model is the following:

5 $$ln(P_{jdt}) = \beta _{0} + \beta _{1} R_{jt}^{r} + \beta _{2} H_{jdt} + \beta _{3} Z_{jt} + \beta _{4} X_{pmt} + \beta _{jdt} ,$$

where the dependent variable ${\mathrm{l}\mathrm{n}(P}_{jdt})$ is a natural logarithm of provider *j*’s price in district *d* in the competitive bidding organized at time *t*. The key variable of our interest is the risk measure ${R}_{jt}^{r}$, which receives value 1 if the provider is located in an area where at least one rejection was done in the previous round, and 0 otherwise. The second empirical model takes the following form:

6 $${\Delta P}_{jdt}= {\beta }_{0}+{\beta }_{1}{R}_{jt}^{d}+{{\beta }_{2}H}_{jdt}+ {{\beta }_{3}Z}_{jt}+{{\beta }_{4}X}_{pmt}+{\varepsilon }_{jdt}$$

where the dependent variable ${\Delta P}_{jdt}$ is the difference in provider *j*’s prices between two competitive biddings. The risk measure ${R}_{jt}^{d}$ in the model is the provider *j*’s distance to the threshold in the previous round, calculated using the quality–price scores. The motivation for explaining price differences with the distance to the threshold is that we are not only interested in the price-level in general, but to investigate whether providers learned that they could have offered higher prices in the previous procurement. In both of the models we control for competition ${H}_{jdt}$, which is calculated using predicted patient flows. We also include vector ${Z}_{jt}$, which controls for providers’ quality, capacity, premises, experience and business type, as well as vector ${X}_{dmt}$, which includes the number of potential patients in municipality *m* as demand-side indicator and rents in postcode *p* as supply-side indicator. These supply and demand-side factors have been shown to influence prices in previous empirical literature [25, 33]. Finally, ${\varepsilon }_{jdt}$ is the error term of the models.

## Results

### Descriptive evidence

Table 1 shows the descriptive statistics of the accepted and rejected providers in the studied competitive biddings. The average price of accepted providers was 33.4 euros in 2003, increased to 39.3 euros in 2006, to 47.5 euros in 2010 and finally to 57.8 euros in 2014. Moreover, the difference between the lowest and highest prices increased from 25 euros in 2003 to 82 euros in 2014. Most of the providers have participated in several competitive biddings, and the proportion of new providers has been around 15% in each procedure.^15^ On average, these experienced providers increased their prices by 5.5 euros 2006, 8.1 euros in 2010 and 10.1 euros in 2014. Providers also increased their annual capacity from an average of 18 annual patients in 2006 to 41 in 2014.^16^ The data show that even though the districts did not implement a systematic capacity-rule for acceptance, the average price of the rejected providers has been higher and quality lower compared to the accepted providers in each procurement. Thus, it seems that the districts only rejected the very few inefficient bids that they received. Also, more than half of the rejected providers were new providers and had a smaller number of existing patients compared to the accepted providers. These providers were most likely the easiest to reject as they had no existing patient relationships.

### Regression results

The main regression results on the effects the risk of rejection has on prices are presented in Table 5. The risk of rejection is measured as whether the provider was located in an area where at least one bid was rejected in the previous competitive bidding. The results show that higher risk had small and statistically weak but negative effects on prices in the 2010 competitive bidding. However, previously made rejections decreased prices by more than 5% in 2014. Thus, the results indicate that rejecting at least one provider maintains a credible risk of rejection and enhances competitive pressure. We have two main explanations why increased risk did not have statistically as strong effects on prices in 2010 as in 2014. First, many of the districts merged between 2006 and 2010, and providers might have anticipated that new geographically larger districts would reject some of the providers. Second, the results indicate that the providers started to learn that the risk of rejection is very low within their district, and this finally actualized in 2014, when providers in areas where no rejections were previously made offered higher prices.

**Table 5:** Regression results: Ln (Price)

	2010			2014		
	Model 1	Model 2	Model 3	Model 1	Model 2	Model 3
Rejections	– 0.004	– 0.028*	– 0.020	– 0.078***	– 0.071***	– 0.054**
	(0.015)	(0.014)	(0.013)	(0.018)	(0.018)	(0.017)
– Ln(Predicted HHI)		– 0.079***	– 0.079***		– 0.034***	–0.026**
		(0.011)	(0.010)		(0.009)	(0.008)
Quality			0.004***			0.008***
			(0.0003)			(0.001)
Premises			0.000			– 0.028
			(0.018)			(0.017)
Capacity			0.0002*			0.0002*
			(0.0001)			(0.0001)
New			0.056***			0.050***
			(0.013)			(0.013)
Rent			0.012***			0.005*
			(0.002)			(0.002)
Potential demand			0.000			0.000
			(0.000)			(0.000)
Business-type	No	No	Yes	No	No	Yes
*N*	1202	1202	1202	1187	1187	1187
*R*^2^	0.0001	0.049	0.278	0.018	0.030	0.196

OLS estimates of Eq. (5) where the dependent variable is Ln(Price)

*HHI* Herfindahl–Hirschman Index

*p < 0.05; **p < 0.01; ***p < 0.001

Table 6 shows the results regarding the effect of providers’ previous distance to the rejection threshold on their price difference between the two competitive biddings. The results show that an increase of one quality–price point from the rejection threshold increased prices by more than 0.15 euros in 2010 and 0.06 euros in 2014. Thus, providers that were further away from the rejection threshold offered higher prices in the next competitive bidding. This indicates that providers learned to evaluate their optimal bids, taken their quality, and noticed that they could take a bigger risk in the next round. Thus, providing providers information regarding their optimal prices and risk of rejection enabled strategic bidding behavior, and increased the overall price-level of the service.

**Table 6:** Regression results: ΔPrice

	2010			2014		
	Model 1	Model 2	Model 3	Model 1	Model 2	Model 3
Distance to threshold	0.165***	0.174***	0.153***	0.073***	0.079***	0.060**
	(0.023)	(0.023)	(0.024)	(0.020)	(0.020)	(0.021)
–Ln (Predicted HHI)		– 1.609***	– 1.607***		– 0.789*	– 0.636
		(0.344)	(0.344)		(0.348)	(0.362)
Quality			0.050***			0.080**
			(0.012)			(0.028)
Premises			– 0.121			– 1.442*
			(0.763)			(0.725)
Capacity			0.001			– 0.001
			(0.004)			(0.004)
Rent			0.178*			– 0.243**
			(0.082)			(0.092)
Potential demand			– 0.0002			0.0003
			(0.0002)			(0.0002)
Business-type	No	No	Yes	No	No	Yes
N	964	964	964	934	934	934
R^2^	0.061	0.082	0.114	0.014	0.019	0.055

OLS estimates of Eq. (6) where the dependent variable is ΔPrice

*HHI* Herfindahl–Hirschman Index

*p < 0.05; **p < 0.01; ***p < 0.001

In both of the regression analyses we also include competition, measured as a negative natural logarithm of a HHI that is based on predicted patient flows. Regarding the analysis on prices (Table 5), the coefficients for competition are negative and statistically significant both in 2010 and 2014 across all of the models. This result indicates that prices were lower in districts that are more competitive. Table 9 (in the Appendix) provides similar regression results using the competition measure based on actual patient flows. This analysis shows that the results regarding the effects of the risk of rejection on prices are robust across the two different measures of market structure. In contrast with the previous work by Pekola et al. [9], our results show that greater competition decreases prices. The difference between the results is most likely due to different measures of competition, as their paper measures the degree of competition using the number of physiotherapy providers in a given municipality.

The main results regarding both of the regression analyses are rather robust across all model specifications, including when we control for various provider and area-level attributes. The results in Table 5 show that providers with greater quality and larger capacity offered higher prices. Also, new providers and providers in more expensive areas offered higher prices. Further, we also tested whether the number of rejections had an effect on prices. These results confirmed that the more providers the district rejected, the lower the price level was in the next round. We also analyzed models including all control variables except quality, because quality and price are likely to be related, even though the measured quality in this study is based on long-term quality investments. This analysis did not change the main results of the study. Finally, we analyzed whether the effect of distance to the threshold was stronger in the districts that made rejections, but found no statistically significant evidence.

## Counterfactual policy analysis

We analyze the effects of implementing a capacity-rule for acceptance in the 2014 competitive bidding for the 2015–2018 contract period. We investigate two capacity-rules that are based on the number of patients in the district added by 10 or 100 percent.^17^ From a practical point of view, the scenarios take into account potential market exits and increasing demand during the contract periods. We perform the analysis using the following three steps: first, we create the counterfactual pools by including all providers in the quality–price lists until the capacity rule is reached.^18^ Second, we examine how patients distribute among providers in the counterfactual pools. We predict the choice of provider among patients who are required to switch, because their initial provider is not included in the counterfactual pool. For the predicted choices we use the estimates from the choice model in 2015 (see Table 4). In these predictions we replace the variable capacity with providers’ remaining free capacity, to take into account that switching patients would be less likely to choose a provider with only little free capacity. The provider with the greatest probability is considered as the chosen one. In the final step we calculate expected annual costs and travel distances per patient at their chosen provider.

Table 7 presents the results of the counterfactual scenarios calculated in 2015. Implementing a 100% capacity-rule results in 586 accepted providers and 601 rejected providers. The average price of the accepted providers is 2.3 euros lower and the average quality 3.6 points higher compared to the actual pools. Because a large number of providers are rejected, 35% of the patients are required to switch providers. However, compared to the actual choices in 2015, an average patient visits a provider with 2.4 points higher quality located only 0.7 km further from their home. Patient-level annual costs are on average 104 euros less than actual costs in 2015, and the total annual costs decrease by 1.35 million euros. This represents annual savings of 2% points. Table 7 also shows estimations for the stricter 10% capacity rule, where only 353 providers are accepted and 60% of the patients are required to switch providers. An average patient visits a provider with 3.7 points higher quality located 3.7 km further away from home, compared to actual choices in 2015. Implementing the rule results in annual savings of 2.3 million euros.

**Table 7:** Results of the counterfactual analysis in 2015

Variable	100% capacity rule						10% capacity rule					
	*N*	Mean	SD	Min	Max	ΔActual	*N*	Mean	SD	Min	Max	ΔActual
Accepted providers												
Price (€)	586	55.50	8.30	37.6	84.5	– 2.33	353	54.66	8.12	37.6	84.5	– 3.17
Quality	586	41.02	5.88	19	55	3.61	353	42.37	5.73	20	55	4.96
Quality–price score	586	78.28	5.56	58.55	100	4.83	353	80.28	5.62	58.55	100	6.83
Rejected providers												
Price (€)	601	60.68	10.54	34	116	– 9.70	834	59.59	10.14	34	116	– 10.79
Quality	601	33.39	7.24	9	50	6.82	834	34.95	7.25	9	50	8.38
Quality–price score	601	67.98	6.71	39.68	83.7	10.83	834	70.01	6.87	39.68	87.29	12.86
Patient characteristics												
Forced switch	13,045	0.35	0.48	0	1	0.35	13,045	0.60	0.49	0	1	0.60
Predicted provider's quality	13,045	43.92	4.88	19	55	2.42	13,045	45.22	4.53	20	55	3.72
Predicted distance (km)	13,045	12.94	20.06	0.5	474.96	0.73	13,045	15.40	22.57	0.5	471.29	3.19
Predicted annual costs (€)	13,045	5020	2937	287.5	24,120	– 104.00	13,045	4951	2907	287.5	23,940	– 173.00
Predicted total costs (€)	65,492,269					– 1,354,849	64,579,814					– 2,267,304

We have predicted the choice of provider for all patients who were forced to switch, because their actual choice was not included in the counterfactual pool of providers, using estimates from the conditional logit choice model (see Table 4). ΔActual is the difference in mean values between actual and counterfactual scenario

Our results show that implementing a capacity-rule would have resulted in large fiscal savings and higher quality of care in the 2015–2018 contract period.^19^ On the other hand, many patients would have been required to switch providers and travel longer distances. Our analysis for the direct fiscal savings can be viewed as lower bound estimates, because rejecting a large number of providers would have probably kept the initial level of the bids lower. Also, the two districts that piloted a fixed price were also excluded from this analysis. Further, the districts have a legal possibility to negotiate direct contracts between patients and providers located in areas with few alternatives. Using these direct contracts can further reduce unnecessary forced switching and increased travel distances in cases where a provider is rejected from the pool of providers but might be necessary for a small portion of patients nearby.

## Discussion

Competitive bidding can be a powerful cost containment mechanism in health services. Even though the use of competitive biddings has increased, there is very little empirical evidence regarding the characteristics of regular competitive biddings in health care. In this study we analyze competitive biddings that were organized repeatedly every 4 years by Kela’s insurance districts to acquire multiple providers for a physiotherapy service in their area. The districts rejected only very few providers because of a lack of financial incentives to use a systematic acceptance rule, and because the districts did not want to terminate established relationships between patients and their usual providers. We analyze whether these features had an effect on providers’ bidding behavior.

Our descriptive analysis regarding the competitive biddings in 2003, 2006, 2010 and 2014 shows that the overall price-level and dispersion of the bids increased heavily during the period. The regression analysis provides further evidence regarding bidding behavior. We show that providers that are located in areas where at least one bid was rejected in the previous competitive bidding offered 5.5% lower prices in the 2014 competitive bidding. Further, we show that providers that were far above the rejection threshold increased their price bids more than providers closer to the threshold. These results indicate that providers learned the institutional features of the competitive bidding and started to behave more strategically. Finally, we perform counterfactual analysis, which shows that using a systematic 100% capacity-rule of acceptance would have resulted in direct fiscal savings of at least 5.4 million euros in the 2015–2018 contract period. Even though rejecting a larger number of providers might have resulted into forced switching of providers and longer travel distances, patients would have also received a higher quality of care.

**Table 8:** Descriptive statistics of the districts with and without rejections in 2006 and 2010

Variable	Districts with rejections					Districts without rejections				
	*N*	Mean	SD	Min	Max	*N*	Mean	SD	Min	Max
2006										
Bidders	5	27.40	12.40	17	46	48	25.81	20.78	2	125
Quality–price threshold	5	67.79	7.04	59.78	77.01	48	67.48	9.51	44.38	93.16
Patients	5	278.60	199.88	107	611	48	234.06	152.60	32	853
2010										
Bidders	3	37.67	10.50	27	48	24	45.38	17.96	6	85
Quality–price threshold	3	71.47	7.37	63.80	78.50	24	60.41	8.06	40	73.5
Patients	3	362.00	142.52	233	515	24	489.50	201.23	49	942

Our study offers some important policy implications in regularly arranged competitive biddings with multiple winners in health care. We show that unless a clear rejection threshold is used, the competitive pressure regarding prices does not exist. Thus, the overall price-level increases when providers eventually learn that the risk of rejection is low. Applying a systematic capacity-rule of acceptance has two main features that increase efficiency of the services. First, the capacity-rule brings positive dynamic effects because the competitive pressure increases. Even though our analysis focuses only on price competition, it is likely that also quality competition increases as a result of a higher quality–price threshold for acceptance. Second, the capacity-rule also has direct effects because only the most efficient providers with the greatest quality–price ratio are accepted in the pool of providers.

**Table 9:** Regression results: Ln (Price)

	2010		2014	
	Model 1	Model 2	Model 1	Model 2
Rejections	– 0.027	– 0.017	– 0.066***	– 0.053**
	(0.014)	(0.013)	(0.018)	(0.017)
–Ln(Predicted HHI)	– 0.078***	– 0.073***	– 0.042**	– 0.028**
	(0.011)	(0.010)	(0.009)	(0.009)
Quality		0.004***		0.008***
		(0.0003)		(0.001)
Premises		0.0003		– 0.022
		(0.018)		(0.018)
Capacity		0.0002*		0.0002**
		(0.0001)		(0.0001)
New		0.058***		0.049***
		(0.013)		(0.013)
Rent		0.012***		0.004
		(0.002)		(0.002)
Potential demand		0.000		0.000
		(0.000)		(0.000)
Business-type	No	Yes	No	Yes
N	1202	1202	1187	1187
R^2^	0.042	0.266	0.035	0.196

OLS estimates of Eq. (5) where the dependent variable is Ln (Price)

*HHI* Herfindahl–Hirschman Index

*p < 0.05; **p < 0.01; ***p < 0.001

The results raise some challenges for practical decision-making. It might be difficult for the service organizer to suddenly reduce the number of accepted providers for two main reasons. First, rejecting providers might end a large number of existing relationships between patients and providers. Second, it is likely that the travel distances would increase as a result of the rejections. In 2018 Kela renewed its procurement practices. Five large districts that organized the procurement were instructed to accept providers based on the number of patients in their area and the capacity of the providers added by around 10%. Thus, the main policy recommendation of this paper was placed into practise. However, Kela did receive a lot of negative feedback. One possible solution to avoid the negative features is to apply a fixed-price and accept all providers that meet the minimum quality standards. This requires information about the supply-side costs to determine the right price-level. A fixed price might also not encourage providers to invest in their quality beyond the minimum necessary level, unless patients have freedom of choice and quality information about the providers.

## Appendix

See Appendix Fig. 3; Tables 8, 9.

## Footnotes

1 This behavior is similar to tacit collusion in repeated auctions [20].

2 Some patients may have received physiotherapy organized by their municipality before Kela accepts their rehabilitation plan or after they turn 66 years and are not entitled to the service organized by Kela.

3 If patients have a referral from the doctor, they are entitled to a small reimbursement paid by Kela from the National Health Insurance.

4 Other large physiotherapy purchasers such as municipalities also use repeated competitive biddings, and there may be spillover effects in the procurements between the different markets because many providers are present in the different markets. However, we do not have data on other procurements and the focus is therefore in the markets organized by Kela.

5 Exceptions are South Ostrobothnia and Päijät-Häme districts that implemented a fixed price pilot in the 2011–2014 contract period.

6 Unfortunately, data on prices prior to 2003 was not available.

7 In 2006, the quality–price scoring rule was quality/price, in 2010 quality was weighted 60% and price 40%, whereas in 2014 quality and price were both weighted 50%.

8 The quality–price score tables have been available from the districts also later on.

9 Unfortunately we do not have data about bids that did not meet the minimum requirements.

10 The 2003 data is based on a list of providers’ accepted prices in November 2006.

11 We use a distance of 0.5 kilometres when patient and provider were located in the same postcode. The smallest distance between two independent postcodes in our data is 0.93 kilometres. Unfortunately, we did not have access to data on actual travel times between postcodes.

12 We acknowledge that the physiotherapy market has many not-for-profit providers.

13 The quality–price score was calculated simply as quality/price in 2006. We have transformed this into a similar quality–price measure as in 2010 and 2014 using weights of 50% for both quality and price. This does not change the original order of the providers in the districts’ score tables and makes the results comparable in the different competitive biddings.

14 Figure 3 (in the Appendix) illustrates the intuition: The highest ranked provider received 45 quality points and offered a price of 58 euros. The provider receives the quality–price scoring table after the procedure and notices that many providers with the same or lower quality points offered a higher price and received a contract. Thus, it is likely that this provider will bid a higher price in the next round compared to a provider with lower rank.

15 Altogether the data includes 2023 different providers, 36% of which participated in all of the four procurements.

16 All of the districts did not include capacity in their quality–price score tables in 2006.

17 In the 2018 competitive bidding, Kela used a capacity-rule based on number of patients in 2017 added by 10 percent, but accepting at least three providers in each municipality to ensure short travel distances.

18 For example the district of Helsinki was responsible for organizing the service for 985 patients in 2014. The district received bids from 72 providers with a total capacity of 3471 in the 2014 competitive bidding for the contract period 2015–2018. The district accepted all 72 providers into the pool. Using the 10 percent capacity-based rule we create a counterfactual pool where the first providers in the quality–price list are accepted until the required capacity of 1084 is reached. In this example, the first 23 providers are accepted in the counterfactual pool and the remaining 49 providers in the list are not included.

19 We performed a similar analysis for the 2010–2014 contract period. The results are similar to the 2015–2018 period, but potential direct fiscal savings would have been lower because the overall price-level of the bids was lower. The results are available from the authors.
